# Dynamics of B-Cell Responses after SARS-CoV-2 Vaccination in Spain

**DOI:** 10.3390/vaccines10101615

**Published:** 2022-09-26

**Authors:** Miriam San José-Cascón, Raquel de la Varga-Martínez, Antonio Campos-Caro, Carmen Rodríguez

**Affiliations:** 1 Immunology Department, Hospital Universitario Puerta del Mar, 11009 Cádiz, Spain; 2 Biomedical Research and Innovation Institute of Cádiz (INiBICA), 11009 Cádiz, Spain; 3 Genetics Area, Biomedicine, Biotechnology and Public Health Department, School of Marine and Environmental Sciences, University of Cadiz, 11510 Cádiz, Spain

**Keywords:** SARS-CoV-2, COVID-19, vaccine immunogenicity, B cell, plasmablast, plasma cell

## Abstract

The high mortality rate due to COVID-19 has necessitated the mass vaccination against SARS-CoV-2 to induce protective humoral and cellular immunity. (1) Objective: To study the dynamics of SARS-CoV-2-specific B cells after two doses of the Pfizer-BioNTech SARS-CoV-2 vaccine. (2) Methods: Immunophenotyping and cellular cultures were used to determine the kinetics of B-cell subpopulations and vaccine responses in volunteers before and seven days, three months and seven months after the second dose in Spain (n = 19). (3) Results: Seven days after immunisation, memory B cells and plasmablasts expressing receptors for factors implicated in the maturation of plasma cells were augmented in blood. Three months after vaccination, SARS-CoV-2 spike-specific plasmablasts disappeared from circulation while spike-specific memory-B cells circulated, with heterogeneous dynamics among individuals. (4) Conclusion: After vaccination, specific plasmablasts equipped with receptors for maturation factors were quickly generated and disappeared rapidly from the blood, while specific memory B cells circulated for at least seven months.

## 1. Introduction

The high mortality rate due to COVID-19 has necessitated the mass vaccination against SARS-CoV-2, which induces protective humoral and cellular immunity [1,2]. The Pfizer-BioNTech SARS-CoV-2 mRNA vaccine BNT162b2 induces maturation of B cells and robust humoral immune responses [3,4,5,6,7,8,9,10,11,12]. The first dose mainly activates a non-neutralising recall response while the second dose boosts the neutralising B cell responses [12]. Therefore, a second dose is vital for protection. The secondary immune response to the mRNA SARS-CoV-2 vaccine generates specific memory B cells (MBCs) and plasma cells (PCs) that are based on the proper development of a germinal centre-dependent humoral responses [5,10,11]. It has been shown that SARS-CoV-2 infection induces bone marrow PCs in humans [13], although less knowledge exists about the efficient generation of long-lived PCs after SARS-CoV-2 mRNA vaccine BNT162b2. The cellular bases of the antiviral humoral immune responses are PCs that produce specific antibodies (Abs). PCs originate after stimulation and maturation of B cells in secondary lymphoid organs. Different subpopulations of B lymphocytes are generated, including MBCs and plasmablasts (PBs), which are exported to the bloodstream. Subsequently, circulating PBs mature into long-lived PCs capable of producing high-affinity Abs in large quantities and over a long period of time. This maturation process takes place in specific niches that produce signals through cell contact and cytokines, chemokines and soluble factors. Stromal cells, megakaryocytes and eosinophils producing IL-6, APRIL, B-cell activating factor (BAFF) and CXCL-12, among other factors, have been identified as components of the PC-specific niches in various organs [14,15,16,17,18,19]. In addition, IL-21 has been shown to act directly on human early PCs and circulating PBs [18,19]. These factors upregulate anti-apoptotic genes and support the life span of both short-lived PCs and long-lived PCs, promoting their long-term survival. Therefore, the differential expression of receptors for those factors in PCs/PBs lead the PCs maturation process and, ultimately, long-term humoral immunity and perdurable protection [16].

The aim of our study was to study the dynamics of SARS-CoV-2-specific B cells after two doses of the Pfizer-BioNTech SARS-CoV-2 mRNA vaccine BNT162b2 [1]. Accordingly, the kinetics of B-cell subpopulations and vaccine responses in a cohort of nonpreviously infected volunteers were determined before and seven days, three months and seven months after the second dose of the vaccine. In addition, the other objective of our study was to determine the expression of niche-specific receptors for long-lived PCs generated after vaccination.

## 2. Materials and Methods

### 2.1. Study Population

Employees of the Immunology Department and Research Unit from Hospital Universitario Puerta del Mar, Cádiz, Spain who received two doses of the Pfizer-BioNTech SARS-CoV-2 vaccine and were not previously infected with COVID-19 were included in the study. Initially, 26 individuals were recruited and started the study. None had left the study by day 7 after vaccination. After three months and seven months, three and four individuals had contracted COVID-19, respectively, and were excluded from the study. Finally, the complete study was carried out with 19 individuals (n = 19, 12 women and 7 men; mean age = 43 ± 13 years, range = 26 to 60). Heparinised blood samples were obtained prior to and seven days, three months and seven months after the second dose of the SARS-CoV-2 vaccine. For the control experiments, blood samples were obtained from healthy donors seven days after receiving a conventional booster immunisation against tetanus toxoid (TT) [17]. Participants were informed of the objective of the study and gave their consent according to the Declaration of Helsinki.

### 2.2. Reagents

The monoclonal Abs (mAbs) and cytokines used in this study for flow cytometry and cell cultures studies are detailed in Table S1.

### 2.3. Distribution of B-Cell Subpopulations and Cytokine and Chemokine Receptor Expression by Circulating PBs

Immunophenotyping was performed by flow cytometry (FC) in a FACSCanto cytometer (Becton Dickinson; San Jose, CA, USA). In flow cytometry studies, complete blood was used for analysing the distribution of B-cell subpopulations in blood (100 µL per test). For immunophenotyping of purified enriched B-cells, cells (1 × 10^6^ µL, 100 µL per test) were incubated in staining buffer (FACS™flow, BD, Catalog No: 342003) with the appropriate amounts of fluorochrome-conjugated antibodies for 15 min in the dark (Table S1). Next, two washes were made with phosphate buffered saline (PBS) (10 mL, centrifuged 7 min at 400× *g*) and resuspended in 300 μL of PBS for subsequent acquisition in a flow cytometer [20]. To discriminate live single cells from debris, dead cells and doublets we used a method based on gating lymphoid cells by using forward scatter (FSC)-Area (FSC-A), FSC-Height (FSC-H) and side scatter (SSC) parameters. Events with low size in FSC vs. SSC dot plot and/or placed outside the forward scatter FSC-A vs. FSC-H plot diagonal were excluded from all gated cell populations. Then, a gate was set to detect CD45+CD19+ cells. PBs were identified as CD19^low^CD20-CD27^high^CD38^high^, naive B cells were identified as CD19+CD20+IgD+CD27-, switched MBCs (SW-MBCs) were identified as CD19+CD20+IgD-CD27+, nonswitched MBCs (NSW-MBCs) were identified as CD19+CD20+IgD+CD27+ and double-negative B cells (DNs) were identified as CD19+CD20+IgD-CD27-. The expression of CD126 and CD130 chains of IL-6R, IL-10R (CD210), IL-21R, and BAFF and APRIL receptors (BCMA and TACI) was determined by the FC of the CD19^low^CD38^high^ PB gate. The percentage of positive cells and the intensity of expression were recorded.

### 2.4. Cell Preparation and Cell Cultures

SARS-CoV-2 spike-specific PBs were functionally identified as cells capable of spontaneously producing anti-SARS-CoV-2 Abs after seven days of culture. Live peripheral blood mononuclear cells obtained by centrifugation on a Hystopaque 1077 gradient and depletion of T lymphocytes by the rosette formation technique with sheep red blood cells to obtain the enriched-B cells fraction [19]. Afterwards, cells were cultured in 96-well flat-bottomed plates in a final volume of 250 μL in RPMI-1640 medium supplemented with glutamine (10 mM), decomplemented fetal calf serum (10%), streptomycin (100 µg/mL) and penicillin (100 U/mL) at 37 °C in CO2 (5%) and humidity saturation oven for a period of 7 days. The concentration of enriched B-cells in culture was 0.5 × 10^6^ cells/mL. The final concentrations of reagents added to cell cultures were as follows: IL-21 (50 ng/mL), BAFF (100 ng/mL), anti-CD40 mAb (1 μg/mL) and cycloheximide (10 μg/mL). After the stipulated time, the supernatants of the cultures were obtained by centrifugation and stored at −20 °C for subsequent quantification of anti-SARS-CoV-2 Abs by ELISA (Euroimmun, Lubeck, Germany). Additionally, as a control, enriched B-cell cultures from three healthy individuals immunised with TT before receiving the SARS-CoV-2 vaccine were performed, and the secretion of anti-SARS-CoV-2 Ab and anti-TT Ab [17] into the supernatants was determined (Table S2).

### 2.5. Anti-SARS-CoV-2 Ab Detection in Sera and in Culture Supernatants

A sandwich ELISA (Euroimmun, Lubeck, Germany) was used for the determination of IgG neutralising spike-specific SARS-CoV-2 Abs. The results in sera were reported as the optical density (OD) of individual samples/OD of the calibrator. Supernatants were tested undiluted, and the results were expressed in absorbance relative units (AUs).

### 2.6. Data Analysis

Significant differences were established by the Mann–Whitney U test or Wilcoxon test for unpaired or paired samples, respectively. *p* values lower than 0.05 were considered statistically significant.

## 3. Results

### 3.1. Total PB and MBC Cells Were Augmented in Blood after SARS-CoV-2 Immunisation

**Figure 1:**
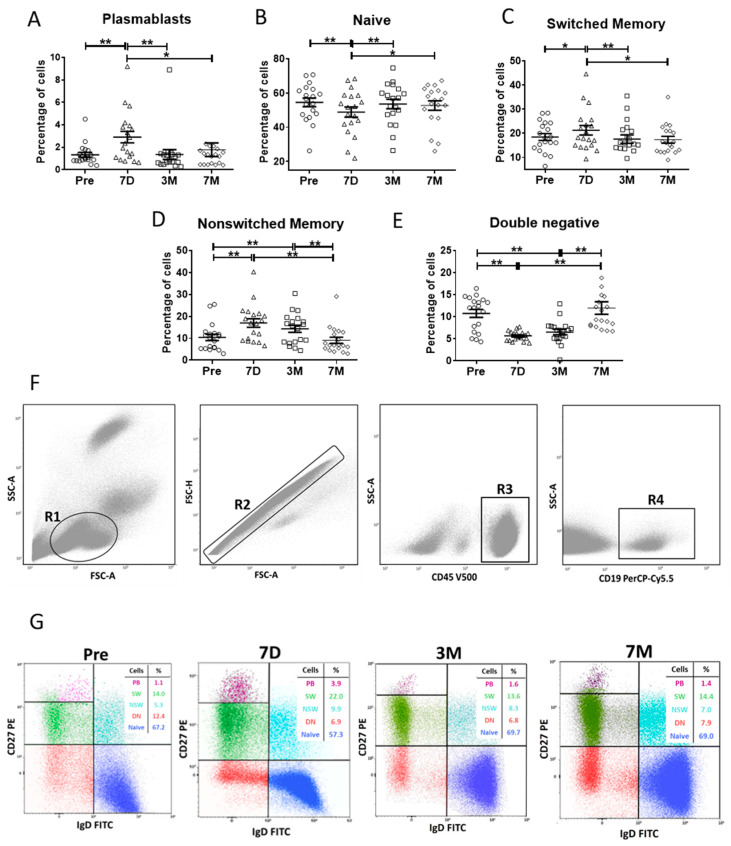
**Distribution of B-cell subpopulations.** (**A**–**E**). Percentages of B-cell subpopulations identified by flow cytometry in the gate of CD45+ CD19+ cells before the first dose of the vaccine (Pre) and seven days (7D), three months (3M) and seven months (7M) after the second dose in 19 individuals. Each data point represents one independent sample. The results are expressed as percentages and represent the mean ± SEM. ** *p* < 0.001, * *p* < 0.05 Wilcoxon test for paired samples. (**A**). Percentage of PB gated as CD19^low^CD20-CD27^high^CD38^high^ (purple). (**B**). Naive B cells gated as CD19+CD20+IgD+CD27- (dark blue). (**C**). SW-MBC gated as CD19+CD20+IgD-CD27+ (green). (**D**). NSW-MBC gated as CD19+CD20+IgD+CD27+ (light blue). (**E**). DN B cells gated as CD19+CD20+IgD-CD27- (red). (**F**). Scheme of gated regions for flow cytometry studies (R1 to R4). (**G**). Representative dot plots from a single experiment are shown. The percentages of each B-cell subpopulation are depicted in the respective tables. DN: double-negative B cells; FSC-A: Forward Scatter-Area; FSC-H: Forward Scatter-Height; NSW-MBC: nonswitched memory B cells; PB: plasmablasts; SEM: standard error mean; SW-MBC: switched memory B cells; SSC-A: Side Scatter-Area.

One week after vaccination, PBs significantly increased (3.1 ± 0.6% vs. 1.4 ± 0.2%, mean ± SEM; *p* < 0.0001, Figure 1A). At the same time, SW-MBC (Figure 1C) and, particularly, NSW-MBC (Figure 1D) were also augmented in blood. Consequently, naive B cells diminished (Figure 1B). By three months after vaccination, PBs, SW-MBCs and naive B cells had returned to basal levels (Figure 1A, Figure 1C, Figure 1B, respectively) while NSW-MBCs remained elevated (Figure 1D). By seven months after vaccination, no significant differences in B-cell subpopulations were observed with respect to preimmunisation levels (Figure 1A–E). Representative dot plots are shown in Figure 1F,G.

### 3.2. Blood PB Generated after Immunisation Express Receptors for Cytokines and Chemokines Implicated in the Maturation of PC

**Figure 2:**
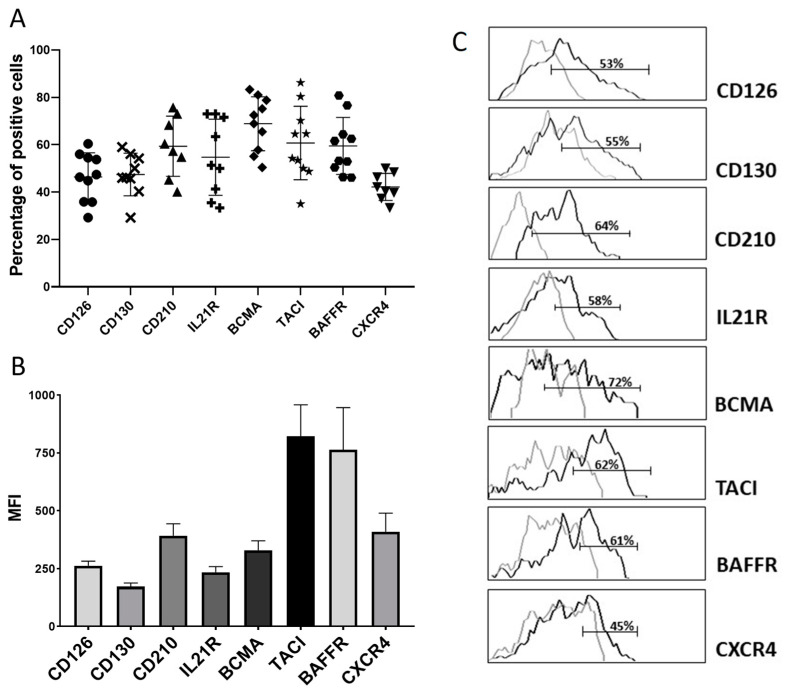
**Cytokine and chemokine receptors expressed in blood PBs after immunisation.** The percentage (**A**) and mean fluorescence intensity (**B**) of CD126, CD130, CD210, IL-21R, BCMA, TACI, BAFFR, and CXCR4 expression were determined by flow cytometry on CD19^low^CD38^high^ PB from the enriched B-cell fraction in vaccinated individuals seven days after the second dose of the vaccine (n = 10). The results are expressed as the mean ± SEM of 10 independent experiments. The mean percentage of positive PBs for each marker is: CD126: 46.4%; CD130: 47.4%; CD210: 59.3%; IL21R: 54.7%; BCMA: 68.8%; TACI: 60.7%; BAFFR: 59.4%; CXCR4: 42.1%. (**C**). Representative histograms are shown. Isotype controls are shown as grey lines. MFI: mean fluorescent intensity; PB: plasmablasts; SEM: standard error mean.

Approximately half of the PBs expanded seven days after the booster expressed both components of the IL-6 receptor complex (CD126 and CD130), the receptors for IL-2 and IL-10 and the CXCL12 receptor CXCR4 (Figure 2A). BCMA and, particularly, BAFFR and TACI, the receptors for BAFF and APRIL respectively, were expressed with high intensity in a higher proportion of PBs (Figure 2A–C). The percentage of PBs in basal conditions represents a minority population that in the absence of stimulation (infection, vaccination, inflammation, autoimmunity, etc.) is practically undetectable in peripheral blood by cytometry [15,17,18,19,20,21]. Therefore, due to the PB scarcity, we were only able to perform the complete PBs’ immunophenotype after vaccination, in ten individuals (Figure 2A,B).

### 3.3. Seven Days after Vaccination, Specific PB-Producing Anti-SARS-CoV-2 Ab Circulate

**Figure 3:**
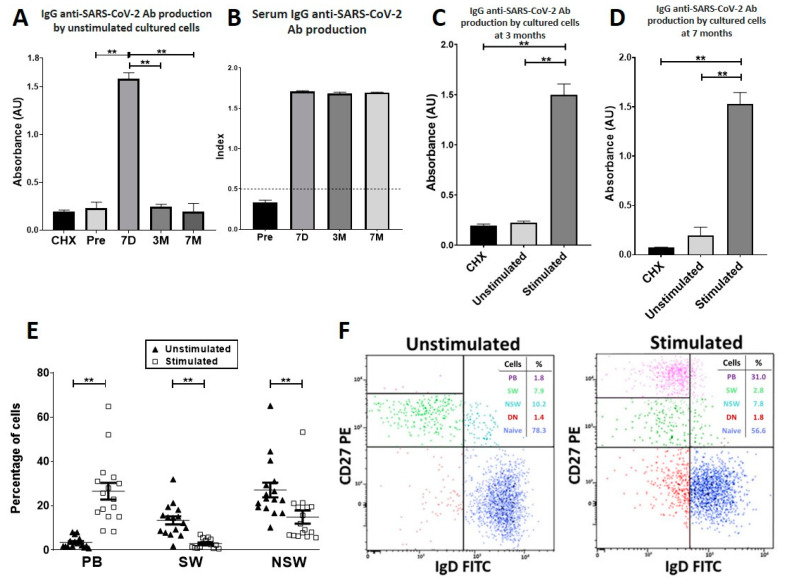
**Specific anti-SARS-CoV-2 Ab responses.** (**A**). IgG anti-SARS-CoV-2 Ab production in unstimulated enriched B-cell cultures before the first dose of the vaccine (Pre) and seven days (7D), three months (3M) and seven months (7M) after the second dose of the vaccine. IgG anti-SARS-CoV-2 Ab production in cultures with CHX at seven days. (**B**). Serum IgG anti-SARS-CoV-2 Ab levels were determined by ELISA. The results are reported as indices. Values above 0.5 were considered positive. (**C**,**D**). IgG anti-SARS-CoV-2 Ab production by cultured enriched B cells at three months (**C**) and seven months (**D**) after the second dose of the vaccine. Stimulated cultures contained a mixture of stimulating anti-CD40 mAb, IL-21 and BAFF. (**E**). B-cell subpopulations in cultures three months after the second dose of the vaccine. Cells collected after the culture period were analysed by flow cytometry. ** *p* < 0.001, Wilcoxon test for paired samples. PB were gated as CD19^low^CD20-CD27^high^CD38^high^ (purple); naive B cells were gated as CD19+CD20+IgD+CD27- (dark blue); SW-MBC were gated as CD19+CD20+IgD-CD27+ (green); NSW-MBC were gated as CD19+CD20+IgD+CD27+ (light blue) and DN cells were gated as CD19+CD20+IgD-CD27- (red) (n = 16). The results are expressed as the mean ± SEM of 16 independent experiments. (**F**). Representative dot plots of B-cell subpopulations in both unstimulated and stimulated cultures three months after the second dose of the vaccine are shown. Ab: antibodies; CHX: cycloheximide; IgG: immunoglobulin G; DN: double negative B cells; mAb: monoclonal antibody; NSW-MBC: nonswitched memory B cells; PB: plasmablasts; SW-MBC: switched memory B cells.

As shown in Figure 3A, the levels of spontaneous secretion of IgG anti-SARS-CoV-2 Abs in cell cultures before stimulation were negligible. In contrast, 7 days after vaccination, a 5.1-fold significant increase was observed. This production could be drastically reduced by the inclusion of the protein-synthesis inhibitor cycloheximide (CHX) in the cell cultures, indicating that the secretory capacity was actively carried out by the cultured cells (Figure 3A) [19]. Anti-TT Abs, but not anti-SARS-CoV-2 Abs, was detected in the supernatants of B-enriched cell cultures from three individuals immunised against TT before receiving the SARS-CoV-2 mRNA vaccine (Table S2). Seven days after vaccination, specific serum Abs were found (Figure 3B).

### 3.4. At Three and Seven Months after Vaccination, Specific PBs Disappeared from Blood, but Specific MBCs Were Able to Differentiate into Circulating Anti-SARS-CoV-2-Producing Antibody-Secreting Cells

Figure 3A shows that at three and seven months after immunisation, no spontaneous production of anti-SARS-CoV-2 Ab was secreted into the supernatants of B-enriched cell cultures of vaccinated individuals, thus indicating that PBs had disappeared from circulation. However, anti-SARS-CoV-2 Ab secretion was induced in stimulated cultures with a mixture of anti-CD40 mAbs plus IL-21 and BAFF [20] (Figure 3C,D, Table S2). Analysis of B cells after the culture period revealed that under stimulation, CD27+ MBC differentiated into CD19^low^CD38^high^ PB (Figure 3E,F). At both three and seven months after vaccination, serum Ab production continued to be positive (Figure 3B).

## 4. Discussion

In our work, we have provided the dynamics of spike-specific B cells capable of producing neutralising antibodies after a booster of Pfizer-BioNTech SARS-CoV-2 mRNA vaccine BNT162b2. Several models of PB development after immunisation have established that a wave of specific PBs is generated seven days after vaccination [14,15,17,18]. Accordingly, we found that seven days after immunisation with two doses of the Pfizer-BioNTech SARS-CoV-2 mRNA vaccine, a robust and significant increase in total circulating PB was detected in all the participants (Figure 1A,G). Total CD27+ MBC were also elevated, particularly the compartment of IgD+CD27+ NSW-MBC cells (Figure 1). Three months after immunisation, only the compartment of IgD+CD27+ NSW-MBC remained elevated, and by seven months after immunisation, the levels of B-cell subpopulations had returned to preimmunisation levels. Our results are in agreement with previous studies that show an increase in the percentage of PB and SW-MBC following the second dose of mRNA vaccine [6,9].

PB migration to niches in deposit organs and consequent maturation into long-lived PCs is known to depend on several chemokines and cytokines, among other factors [14,15,16,17,18,19]. We found that these PBs were armed with receptors for maturation-related factors because they expressed both components of the functional IL-6 receptor complex (CD126 and CD130), IL-21R, IL-10R, CXCR4, and, particularly, the BAFF and APRIL receptors BAFFR, TACI and BCMA. This expression profile is similar to that of PB generated after immunisation with TT, although levels of expression for BAFF and APRIL receptors predominated over IL-6 receptor expression in PB generated after SARS-CoV-2 mRNA vaccination [18]. These differences in the receptor expression profile in PB could be due to the type of vaccine we have used in each case: mRNA vaccine (SARS-CoV-2 mRNA BNT162b2S) and protein vaccines (plus adjuvants) in the case of the TT vaccine.

To determine the presence of circulating spike (S)-specific PBs after vaccination, a functional approach was used [18,19,21]. As PBs/PCs are characterised by secreting active and spontaneous Abs [15], blood-enriched B-cell cultures were performed without stimulation. We identified the presence of anti-S PBs in those individuals whose blood-enriched B-cell nonstimulated cultures contained anti-S Abs, as a difference from those cultures before immunisation that did not contain anti-S Abs (Figure 3A, Table S2). In addition, anti-S Ab secretion into the cultures after immunisation was active (Figure 3A). To confirm the specificity of the method, three individuals were vaccinated against TT before receiving the SARS-CoV-2 mRNA vaccine, and unstimulated cultures of enriched B cells were performed seven days after TT vaccination. Although anti-TT Abs were secreted into the supernatants, they did not contain anti-S Abs (Table S2). By using this functional approach, our results indicate that specific PBs circulated seven days after immunisation in every individual included in the study (Figure 3A, Table S2). At that moment, anti-S Abs were also detected in the sera of every participant (Figure 3B). At three and seven months after immunisation, S-specific PB had disappeared from circulation in all the subjects (Figure 3A). These results agree with others that show that immunisation of humans with the Pfizer-BioNTech SARS-CoV-2 mRNA vaccine induces a robust but transient circulating PB response [10]. Serum anti-S Abs stayed positive (Figure 3B), indicating that this production was dependent on PCs located in niches or on the differentiation of MBCs into Ab-secreting cells.

Next, we focused on S-specific MBCs. S-specific MBCs capable of differentiating into anti-SARS-CoV-2-producing antibody-secreting cells circulated (Figure 3 and Table S2), and they were identified in cultures of enriched B cells that contained anti-S Abs after B-cell stimulation (anti-CD40 stimulating mAb plus IL-21 and BAFF) [20]. In fact, PBs increased at the expense of MBC differentiation in stimulated cultures. We found that S-specific MBCs continued circulating at three and/or seven months after vaccination, although, interestingly, the kinetics varied among individuals (Table S2). The reason for the differences on MBC recirculation among participants is unknown, and we did not observe significant variations in serum anti-SARS-CoV-2 Abs among them (data not shown). This variability could reflect individual immunological conditions or responses to vaccination. A consistent and long-lasting S-specific MBC response was detected in most individuals (14 out of 19) and was detected both at three and seven months after vaccination. This fact can be related to the prolonged germinal centre response documented after SARS-CoV-2 mRNA BNT162b2S vaccination [10,11]. The other three individuals (#10, #12, #16) showed a low or absent stimulated production of anti-S Abs in culture at three months, although the value increased by seven months, suggesting that in those individuals, the generation or exportation of MBCs from the germinal centre was delayed [22]. On the other hand, in two individuals (#1 and #7), S-specific MBC was detected at three months after immunisation but had disappeared at seven months, thus indicating a shorter recirculation period and suggesting a rapid migration to deposit organs or differentiation into specific PCs. This altogether suggests that unknown factors could be modulating the temporality in the generation/migration of PBs to the final deposit niches. A limitation of our study is that Omicron spike/receptor-binding domain specific memory B cell flow cytometry data and Omicron specific antibody titres were not performed. It is important to determine the effectiveness of vaccines against Omicron variants as there is a risk for vaccine effectiveness, although most studies have suggested that vaccines are still effective against the currently circulating variants [23]. On the other hand, multiple variants caused by genetic mutations or viral recombination, with distinct antigenic expression of spike protein, have emerged throughout this pandemic, peaking and disappearing quickly (https://www.ecdc.europa.eu/en/covid-19/variants-concern; https://www.cdc.gov/coronavirus/2019-ncov/variants/variant-classifications.html, accessed on 10 September 2022), and, unfortunately, the future variants of SARS-CoV-2 are unpredictable. The role of spike protein is common to all of them, namely, in the invasion of host cells. Therefore, our results indicating that the secondary immune response to mRNA SARS-CoV-2 vaccine rapidly generates spike-specific experienced B cells and PBs are valuable [5,10,11].

We show that, after the booster, spike-specific PBs rapidly circulated and promptly migrated from circulation, possibly to resident areas and niches for ulterior maturation, as they were equipped with niche-related receptors. Their potential role is in becoming long-lived PCs capable of producing anti-spike Ab for long periods of time and, finally, for long-term humoral immunity. More studies are necessary to determine their transcriptional profile and the requirements for the establishment of a stable pool in the bone marrow [15,16,17].

Furthermore, we found that spike-specific MBCs able to differentiate into spike-specific PBs recirculated for at least seven months in most individuals. They patrol and can be stimulated by new infections, therefore contributing to maintaining the long-term MBC cell pool. MBC can rapidly modulate the Ab binding affinity of their receptors by somatic mutation mechanisms, potentially generating higher-affinity Ab in subsequent infections or boosters. This could apply to Omicron and newer variants based on spike protein mutations. Although a controversial issue emerged for the convenience of repeated booster shots, they have been shown to reduce severe COVID-19 (https://www.cdc.gov/media/releases/2022/s0901-covid-19-booster.html, accessed on 10 September 2022), probably by generating new waves of MBC and PB with adapted affinity against new spike-protein variants. Other strategies for SARS-CoV-2 vaccination are being developed, such as heterologous vaccination, multivalent vaccines, mucosal vaccines, etc. [23]. The immunogenicity and the effectiveness of these new vaccination strategies remain to be established.

## 5. Conclusions

In conclusion, our results indicate that after the second dose of Pfizer-BioNTech SARS-CoV-2 mRNA vaccine BNT162b2, a wave of PBs equipped with receptors necessary for maturation into long-lived PCs is quickly exported to circulation. SARS-CoV-2-specific PBs disappear rapidly from the blood. SARS-CoV-2-specific MBCs are also generated and circulate in most individuals for at least seven months, with a heterogeneous response in each individual, both in terms of appearance in blood and in exportation to resident tissues. Serum-specific IgG antibodies persist for at least seven months, suggesting that they originate from long-lived PCs residing in deposit areas or from MBCs that differentiate into specific antibody-secreting cells.

## Data Availability

Data available upon request.

## Supplementary Materials

The following supporting information can be downloaded at: https://www.mdpi.com/article/10.3390/vaccines10101615/s1, Table S1: Monoclonal antibodies and cytokines used in this study. Table S2: Anti-SARS-CoV-2 Ab production in enriched B-cell cultures from SARS-CoV-2 vaccinated individuals and from controls (* tetanus toxoids vaccinated, non-SARS-CoV-2 vaccinated).
