# Supplementary material for: Dynamics of B-Cell Responses after SARS-CoV-2 Vaccination in Spain
Source: Vaccines (Basel). 2022 Sep 26;10(10):1615. doi: 10.3390/vaccines10101615 (PMC9608951; doi:10.3390/vaccines10101615)
Supplement: Supplementary file 1 [file vaccines-10-01615-s001.zip › vaccines-1894444-supplementary.pdf]

Table S1: Monoclonal antibodies and cytokines used in this study

Antibody	Fluorochrome	Brand	Clone	Concentration	Vol. Per Test	Cat. number
Anti-CD19	PerCP-Cy5.5	BD	SJ25C1	5 μg/mL	20 μL	332780
Anti-CD19	APC	BD Pharmingen	HIB19	0.2 mg/mL	20 μL	555415
Anti-CD38	FITC	BD	HB7	6 μg/mL	20 μL	340927
Anti-CD38	PE-Cy7	BD	HB7	25 μg/mL	5 μL	335790
Anti-IgD	FITC	BD Pharmingen	IA6-2	0.5 mg/mL	20 μL	555778
Anti-CD20	V450	BD	L27	100 μg/mL	5 μL	655872
Anti-CD45	V500	BD	HI30	100 μg/mL	5 μL	560777
Anti-CD27	PE	BD	L128	1.6 μg/mL	20 μL	340425
Anti-CD126	PE	BD Pharmingen	M5	0.5 mg/mL	20 μL	551850
Anti-CD130	PE	BD Pharmingen	AM64	0.5 mg/mL	20 μL	555757
Anti-CD210	PE	BD Pharmingen	3F9	0.5 mg/mL	20 μL	556013
Anti-CD360 (IL21R)	PE	Biolegend	2G1-K12	50 μg/mL	20 μL	347805
Anti-CD267 (TACI)	PE	BD Pharmingen	1A1-K21-M22	0.2 mg/mL	5 μL	558414
Anti-CD268 (BAFFR)	PE	Biolegend	11C1	50 μg/mL	5 μL	316905
Anti-BCMA	PE	R&D Systems	Polyclonal	NA	10 μL	FAB193P
Anti-CD184 (CXCR4)	PE	BD Pharmingen	12G5	0.2 mg/mL	20 μL	555974
Anti-Mouse $IgG_{1,k}$	PE	Biolegend	MOPC-21	0.2 mg/mL	5 μL	400113
Anti-Rat $IgG_{2a,\kappa}$	PE	BD Pharmingen	R35-95	0.2 mg/mL	5 μL	553930
Anti-Rat $IgG_{1, k}$	PE	BD Pharmingen	R3-34	0.2 mg/mL	20 μL	559318
Anti-Goat IgG	PE	R&D Systems	Polyclonal	NA	10 μL	IC108P
Anti-Mouse IgG_1	PE	BD	X40	50 μg/mL	20 μL	345816
IL-21	NA	Pepro-Tech	NA	50 ng/mL	NA	200-21-B
BAFF	NA	Pepro-Tech	NA	100 ng/mL	NA	310-13
Anti-CD40	NA	Bio-Legend	G28.5	1 μg/mL	NA	303611

Allophycocyanin (APC), allophycocyanin-hilite 7 conjugate (APC-H7), fluorescein isothiocyanate (FITC), Not applicable (NA), peridinin chlorophyll protein-Cy5.5 (PerCP-Cy5.5), phycoerythrin (PE), phycoerythrin-cyanine 7 conjugate (PE-Cy7), violet-FluorTM 450 (V450), violet-FluorTM 500 (V500).

Table S2. Anti-SARS-CoV-2 Ab production in enriched B-cell cultures from SARS-CoV-2 vaccinated individuals and from controls (* tetanus toxoids vaccinated, non-SARS-CoV-2 vaccinated)

Individual ID	Spontaneus production pre-vaccination (Absorbance)	Spontaneus production post-vaccination (Absorbance)	Stimulated production 3 moths post-vaccination (Absorbance)	Stimulated production 7 moths post-vaccination (Absorbance)	Controls*	Anti-SARS-CoV-2 Ab (Absorbance)	Anti-TT Ab (ng/ml) (Positive > 50)
1	0.296	1.471	1.732	0.068	A*	0.071	587 (+)
2	0.223	1.146	1.76	1.693	B*	0.083	425 (+)
3	0.195	1.76	1.716	1.719	C*	0.090	630 (+)
4	0.249	1.777	1.723	1.688			
5	0.593	1.762	1.745	1.676			
6	0.409	1.785	1.698	1.697			
7	0.234	1.699	1.703	0.052			
8	0.643	1.768	1.73	1.651			
9	0.262	1.791	1.709	1.685			
10	0.342	1.759	0.654	1.72			
11	0.429	1.775	1.704	1.712			
12	0.311	1.771	0.541	1.716			
13	0.371	1.773	1.717	1.747			
14	0.579	0.929	1.73	1.707			
15	0.54	0.926	1.721	1.692			
16	0.571	1.219	0.23	1.704			
17	0.335	1.774	1.758	1.697			
18	0.165	1.754	1.752	1.727			
19	0.372	1.761	1.743	1.679			

Ab: antibody; TT: tetanus toxoid.
